# Supplementary material for: Content-rich biological network constructed by mining PubMed abstracts
Source: BMC Bioinformatics. 2004 Oct 8;5:147. doi: 10.1186/1471-2105-5-147 (PMC528731; doi:10.1186/1471-2105-5-147)
Supplement: Additional File 5 — The original Chilibot query results of the term "long-term potentiation (LTP)" and 22 other terms, limiting the latest references analyzed to the years 1990, 1995, 2000, and 2004. [file 1471-2105-5-147-S5.bz2 › chilibotAdditionalFile5/ltp2004/html/NMDA_ACTIN.html]

 


 **NMDA** and **ACTIN** 
  
Found 86 abstracts in PubMed,  **30 abstracts were retrieved and analyzed**.  


---

 Search Google  |
 PDF files only 
|  EDU domain only 

---

**Interactive relationship** (e.g. stimulation, inhibition, etc)

- These results suggest that, in addition to electrophysiological changes,  **NMDA**  receptor activation initiates changes in the  **actin**  cytoskeleton of dendritic spines that stabilize synaptic structure.  Ref: 14555951 Nat Neurosci, 2003
- The glutamate induced declustering of Cupidin preceded that of F  **actin**  and drebrin, was elicited by  **NMDA**  receptor mediated calcium influx, and was followed by a downstream pathway including MAPK ERK and protein tyrosine kinase.  Ref: 14511114 J Neurochem, 2003
- N methyl d aspartate  **NMDA**  receptors regulate structural plasticity by modulating  **actin**  organization within dendritic spines.  Ref: 12857875 Mol Biol Cell, 2003
- ... dispersal of  **NMDA**  receptors occurred by a different mechanism independent of  **actin**  or CaMKII.  Ref: 11896155 J Neurosci, 2002
- The  **NMDA**  receptor antagonist AP 5 impaired the dendritic localization of ZBP1 and beta  **actin**  mRNA and inhibited the KCl induced transport of ZBP1.  Ref: 12716932 J Neurosci, 2003
- For example,  **NMDA**  receptors NMDARs are linked to several proteins in the PSD, such as PSD 95, and are also tethered via binding proteins such as alpha actinin directly to filamentous  **actin**  of the cytoskeleton.  Ref: 11606635 J Neurosci, 2001
- This increase in F  **actin**  content is dependent on  **NMDA**  receptor activation and involves the inactivation of  **actin**  depolymerizing factor cofilin.  Ref: 12741991 Neuron, 2003

**Parallel relationship** (e.g. studied together, co-existance, homology, etc.)

- Localization of  **NMDA**  receptor clusters in dendrites was studied by co transfection of CFP  **actin**  and GFP NR2 subunits followed by anti GFP surface staining.  Ref: 11897109 Neuropharmacology, 2002
- Multiple spatiotemporal modes of  **actin**  reorganization by  **NMDA**  receptors and voltage gated calcium channels.  Ref: 12391325 Proc Natl Acad Sci U S A, 2002
- Regulation of A kinase anchoring protein 79 150 cAMP dependent protein kinase postsynaptic targeting by  **NMDA**  receptor activation of calcineurin and remodeling of dendritic  **actin** .  Ref: 12177200 J Neurosci, 2002
- ProSAPs Shanks are multidomain proteins that interact directly or indirectly with receptors of the postsynaptic membrane including  **NMDA**  type and metabotropic glutamate receptors, and the  **actin**  based cytoskeleton.  Ref: 12065602 J Neurochem, 2002
- Together, p250GAP is likely to be involved in  **NMDA**  receptor activity dependent  **actin**  reorganization in dendritic spines.  Ref: 12857875 Mol Biol Cell, 2003
- AKAP79 150 postsynaptic localization was rapidly regulated by  **NMDA**  receptors through CaN activation and F  **actin**  remodeling, further suggesting that AKAP79 150 signaling scaffold targeting depends on  **actin**  dynamics.  Ref: 12177200 J Neurosci, 2002
- Using primary neurons expressing GFP tagged proteins, we found that profilin, a regulator of  **actin**  polymerization, is targeted to spine heads when postsynaptic  **NMDA**  receptors are activated.  Ref: 14555951 Nat Neurosci, 2003
- In response to synaptic stimulation and  **NMDA**  receptor activation, cortactin redistributes rapidly from spines to dendritic shaft, correlating with remodeling of the  **actin**  cytoskeleton, implicating cortactin in the activity dependent regulation of spine morphogenesis.  Ref: 14684878 J Neurosci, 2003
- The ProSAP Shank family of multidomain proteins of the postsynaptic density PSD can either directly or indirectly interact with  **NMDA**  type and metabotropic glutamate receptors and the  **actin**  based cytoskeleton.  Ref: 12421375 J Neurochem, 2002
- Regulation of  **NMDA**  receptor activity by F  **actin**  and myosin light chain kinase.  Ref: 11606635 J Neurosci, 2001
